# Optimization of Synthesis Conditions of Carbon Nanotubes via Ultrasonic-Assisted Floating Catalyst Deposition Using Response Surface Methodology

**DOI:** 10.3390/nano8050316

**Published:** 2018-05-09

**Authors:** Narges Mohammadian, Seyyed M. Ghoreishi, Samira Hafeziyeh, Samrand Saeidi, Dionysios D. Dionysiou

**Affiliations:** 1 Department of Chemical Engineering, Isfahan University of Technology, Isfahan 84156-83111, Iran; narges.mohammadian@gmail.com (N.M.); samira.hafeziyeh@gmail.com (S.H.); 2 Technische Thermodynamik, Universität Bremen, Badgasteiner Str. 1, 28359 Bremen, Germany; samrandsaidi@gmail.com; 3 Environmental Engineering and Science Program, Department of Chemical and Environmental Engineering, University of Cincinnati, Cincinnati, OH 45221-0012, USA

**Keywords:** floating catalyst deposition, carbon nanotubes, response surface methodology

## Abstract

The growing use of carbon nanotubes (CNTs) in a plethora of applications has provided to us a motivation to investigate CNT synthesis by new methods. In this study, ultrasonic-assisted chemical vapor deposition (CVD) method was employed to synthesize CNTs. The difficulty of controlling the size of clusters and achieving uniform distribution—the major problem in previous methods—was solved by using ultrasonic bath and dissolving ferrocene in xylene outside the reactor. The operating conditions were optimized using a rotatable central composite design (CCD), which helped optimize the operating conditions of the method. Response surface methodology (RSM) was used to analyze these experiments. Using statistical software was very effective, considering that it decreased the number of experiments needed to achieve the optimum conditions. Synthesis of CNTs was studied as a function of three independent parameters viz. hydrogen flow rate (120–280 cm^3^/min), catalyst concentration (2–6 wt %), and synthesis temperature (800–1200 °C). Optimum conditions for the synthesis of CNTs were found to be 3.78 wt %, 184 cm^3^/min, and 976 °C for catalyst concentration, hydrogen flow rate, and synthesis temperature, respectively. Under these conditions, Raman spectrum indicates high values of (*I*_G_/*I*_D_), which means high-quality CNTs.

**Highlights:**

- Floating catalyst (FC) chemical vapor deposition (CVD) method was used to synthesize carbon nanotubes.
- Ultrasonic bath was used in the FC method. It helped to control the cluster size, decrease the produced amorphous carbon, and improve the quality of produced carbon nanotubes (CNTs).
- Response surface methodology (RSM) was utilized as the basis of designing experiments to optimize the operating conditions of the method, which was newly used to synthesize CNTs.
- According to the analysis results, hydrogen flow rate and temperature are more effective parameters than catalyst concentration in this method.

## 1. Introduction

Nanotechnology is a multidisciplinary field dealing with a variety of materials produced at the nanometric scale through various physical, chemical, and biological procedures. The characteristics observed in nanomaterials are different from those of their bulk counterparts. Thus, they may overcome the limitations of existing products in terms of cost, functionality, fabrication strategies, and overall performance. Nanomaterials have been studied in depth as carriers in modern healthcare applications due to their tunable surface characteristics. Spherical nanoparticles are the most practical form of nanomaterials. This is due to the fact that they are easier to manufacture in comparison with nonspherical nanoparticles, such as nanowires, microtubes, and nanotubes. Particularly, carbon nanotubes (CNTs) have a large number of interesting characteristics in terms of structure, morphology, functionality, stability, ease of modification, and appropriateness in hybrid materials [1]. As such, CNTs are often a good candidate for the fabrication of devices with novel features.

CNTs are hollow cylindrical tubes consisting of carbon (graphite) with a high aspect ratio (B1000) and sp^2^ hybridization. Considering the number of graphite layers, CNTs are classified as single-walled nanotubes (SWNTs), double-walled nanotubes (DWNTs), and multiwalled nanotubes (MWNTs) [2,3]. These materials potentially have applications in many areas, such as electronic, mechanical, and gas storage fields [4], photonics, renewable energy, drug delivery, and the biomedical sector [5]. Consequently, CNTs potentially have various applications in several fields, such as effective hydrogen storage media, quantum wires, field emitters, field effect transistors, nanoreactors, field emission displays, adsorbents to control environmental pollution [6,7], and fillers in composites. Hence, CNT production has recently been on a huge increase.

**Figure 1:**
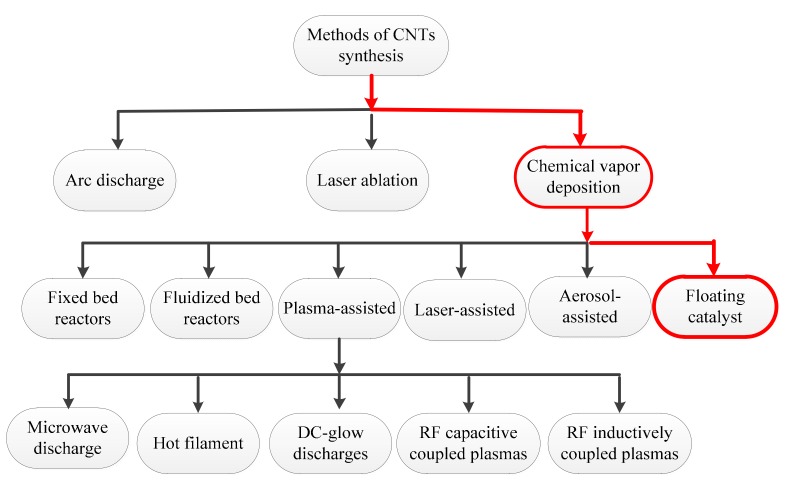
Classification of synthesis methods employed for carbon nanotubes (CNTs).

Various techniques, including arc discharge, laser ablation, and chemical vapor deposition (CVD), can be used to synthesize CNTs. Considering the progress in the laser ablation and electrical arc discharge techniques, significant advancement has been made in large-scale production of CNTs. However, CNT synthesis via these methods cannot be carried out continuously. Thus, the production yield is limited. Furthermore, the products generally contain many impurities, which include amorphous carbon, carbon nanoparticles, C_60_, and other fullerenes [8,9]. Furthermore, arc discharge and laser ablation techniques need high temperatures (>1700 °C) during synthesis. However, they have now been replaced with CVD, which can be conducted at lower temperatures (<800 °C). In addition to these techniques, some nonstandard techniques, such as pyrolysis and hydrothermal treatment, have also been used [2]. Major synthesis strategies for CNTs are briefly shown in Figure 1. 

The synthesis strategy used in the present study is displayed in Figure 1 by the red path. The CVD method was introduced in 1993 for large-scale production of CNTs [10]. It requires the pyrolysis of hydrocarbons (e.g., acetylene, ethylene, propylene, methane, benzene, and toluene) [11,12] or other carbon feedstock (e.g., polymers and carbon monoxide), which are carried in a stream of an inert gas into a chamber with metal catalysts (e.g., Ni, Fe, and Co). A vast variety of parameters (including hydrocarbon precursor composition, catalyst, temperature, pressure, gas flow rate, deposition time, and reactor geometry) play a role in controlling the growth mechanism of CNTs [13,14]. 

The temperature of the CVD-based CNT synthesis generally ranges from 500 to 1200 °C at atmospheric pressure. The basic structure of CNTs (e.g., diameter, length, and alignment) is highly dependent on and can be controlled by controlling the temperature. Moreover, adjusting the gas flow rate can control the pressure of the gaseous carbon precursor. In the case of utilizing a solid hydrocarbon precursor, the vapor pressure is optimized by controlling the hydrocarbon precursor mass, vaporizing temperature, and the carrier gas flow rate. Similarly, for a liquid hydrocarbon precursor, the vapor pressure is optimized by heating the precursor at a specific temperature before being pumped into the reactor. According to Figure 1, several CVD alternatives have been developed for synthesizing CNTs in a controlled way in order to help mass production: (1) fixed bed reactors; (2) fluidized bed reactors; (3) laser-assisted; (4) plasma-assisted (plasma sources including microwave discharge, hot filament, dc-glow discharges, radio frequency (RF) capacitive coupled plasmas and RF inductively coupled plasmas); (5) aerosol-assisted; and (6) floating catalysts [2,15,16]. Recently, the floating catalyst (FC) approach has been the focus of CNT synthesis by some researchers. This is due to its simple equipment, low reaction temperature, low cost, and its ability to produce highly pure CNTs continuously [8,9]. Considering that the FC method has the potential to act as an industrial process for high-yield production of pure CNTs, some investigations have been carried out to optimize the processing operating conditions. It has been found that the decrease in ferrocene/benzene mole ratio contributes to an increase in the diameter of CNTs [4].

Care should be taken for the removal of metal catalyst impurities for any method used to synthesize CNTs because metals may impact the electrocatalytic characteristics of CNTs. Despite the fact that several purification methods are considered to be acceptable, the purified amount of CNTs still varies for different techniques. Large-scale production of purified CNTs needs economic methods for isolating the purified product from metallic and amorphous impurities. It is anticipated that such approaches will decrease the market price of CNTs for application in diverse fields. Therefore, producing CNTs with economic feasibility is a challenge for researchers.

Li and coworkers [17] proposed a possible explanation of the difference in carbon vapor deposition products based on different pyrolytic behaviors of carbon precursors and formation mechanism. Yamada et al. [18] proved that the number of layers in the CNTs depends more on the catalyst size than on the diameter of CNTs. Less amorphous carbon and CNTs with smaller diameter are desired, considering their higher quality. Therefore, controlling the cluster size and consequently their uniform distribution are of high importance because they decrease the tendency of clusters to become agglomerated. 

In this research, a modification was carried out in the synthesis process in order to overcome the shortcomings with respect to the control of cluster size and uniform distribution in the FC method. In this modification, the catalyst was dissolved in xylene outside the reactor, and ultrasonic bath was used to prevent the agglomeration of clusters. It is worth mentioning that using ultrasonic bath hinders the agglomeration. It has been used in different studies for different substances [19,20] and we used it for synthesizing CNTs. According to Beltowska-Lehman et al. agglomeration of nanoparticles can be prevented considerably by physical dispersion, including by ultrasound. The propagation of ultrasonic waves in the bath generates high pressure, inducing stress, which leads to less agglomeration of nanoparticles [21]. According to Versteeg et al. using an ultrasonic atomizing nozzle ensures that the liquid solution is broken up into a fine mist of tiny droplets, which hinders the agglomeration of particles [22]. In order to study the impact of main synthesis parameters and obtain optimized process variables, response surface methodology (RSM) under DESIGN EXPERT software was used. RSM is a collection of statistical and mathematical techniques used for modeling and analyzing engineering problems [23]. In this methodology, the major goal is to optimize the response surface, which is affected by different process parameters at the same time, by considering any possible interactions between different parameters. Moreover, RSM quantifies the relationship between the controllable input parameters and the obtained response surfaces [24].

## 2. Experimental Procedures

### 2.1. Dispersing Catalyst in Carbon Precursor

**Figure 2:**
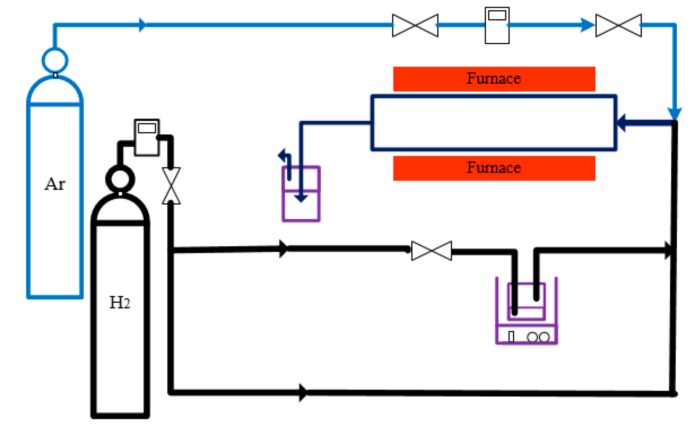
Diagram of the CNT synthesis apparatus.

The old FC method, which is based on dissolving the catalyst in carbon feedstock outside the reactor, exhibits some problems (e.g., difficulty in controlling the size of clusters and their agglomeration). Thus, ultrasonic-assisted CVD method was used in this study to keep the solution homogeneous outside the reactor. In the present work, an apparatus was designed aiming to better control experimental parameters and to obtain CNTs with more desirable properties. The schematic diagram of the apparatus is presented in Figure 2. The quartz tube was 1000 mm long and its diameter was 25 mm. The quartz tube was located in a cylindrical furnace (Carbolite, Watertown, MA, USA). Xylene, hydrogen, and ferrocene were used as the carbon source, the carrier gas, and the catalyst precursor, respectively. It is worth mentioning that the inert gas of argon, which is shown in the Figure 2, was used to remove the oxygen from the reactor.

In order to keep the solution homogeneous, two methods were compared in this study: (1) using stirring heater; and (2) using ultrasonic bath. The obtained results of thermogravimetric analysis (TGA) showed that using ultrasonic bath was a more suitable technique. The ferrocene-xylene solution was preheated at 50 °C. Subsequently, hydrogen gas carried the solution into the reactor. Partial pressure of catalyst/carbon depended on hydrogen flow rate. Using ultrasonic bath (Bandelin Sonorex Super, Berlin, Germany) provided a homogeneous solution in which controlling the ferrocene/xylene proportion was possible. It is worth mentioning that ferrocene and xylene were both provided from Merck KGaA, Darmstadt, Germany. First, the metal-organic precursor was reduced by hydrogen to form atomic iron and agglomerated to iron clusters or iron particles to grow CNTs. After CNT synthesis, the carbon products were deposited on the cooler zone of reaction tube wall.

In order to characterize the morphology and microstructure of the synthesized products, some characterization techniques were applied, including: TGA, field-emission scanning electron microscopy (FESEM), transmission electron microscopy (TEM), and Raman spectroscopy.

### 2.2. Experimental Design and Data Analysis

RSM is an effective and flexible experimental design methodology for modeling and analyzing a problem in which the response is under the influence of several variables [25,26]. After performing the designed experiments, the procedure of response analysis is carried out and the presented model is analyzed by general statistical tests, such as Fisher’s statistical test (F-test). The test determines the F-value, the *p*-value, and R^2^ coefficient and the model can be analyzed by investigating these values. F-test can state the importance of any process parameter. The larger the F-value, the more valid the model [27]. For any parameter to be a significant, the *p*-value (probability value) must be less than 0.05. However, in RSM, a *p*-value of lack-of-fit >0.05 (not significant) means that the model fits well [28]. The coefficient of determination (R^2^ value) certifies reasonable fitting of the model with experimental data. If R^2^ is close to 1, it shows good fitness of the correlation between the predicted and experimental values [29].

In comparison with the conventional optimization method, RSM can provide more information from fewer experiments. Hence, it is time-saving and economic. [30]. RSM consists of a collection of empirical techniques, which are allotted to the evaluation of the relationship that exists between a cluster of controlled experimental factors and measured responses based on one or more chosen criteria. In RSM, central composite design (CCD) determined the optimum operating conditions for the synthesis of CNTs [31]. Optimization studies were performed by studying the impact of three variables: hydrogen flow, catalyst concentration, and synthesis temperature. The system behavior is explained by the following empirical second-order polynomial model (Equation (1)):
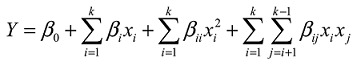
(1)
where *Y* is the predicted response, and *x_i_*, *x_j_*, …, *x_k_* represent the input variables, which influence the response *Y*. *x_i_*^2^, *x_j_*^2^, …, *x_k_*^2^ are the square effects, *x_i_x_j_, x_i_x_k_*, and *x_j_x_k_* are also the interaction effects. *β*_0_ is the intercept term, *β_i_*(*i* = 1, 2, …, *k*) is the linear effect, *β_ii_* (*i* = 1, 2, …, *k*) is the squared effect, and *β_ij_* (*i* = 1, 2, …, *k*; *j* = 1, 2, …, *k*) is the interaction effect [32].

The DESIGN EXPERT software (Design Expert 6, Stat-Ease, Minneapolis, MN, USA) was used for regression and graphical analysis of the obtained data. In this research, CCD was utilized to probe the characterization of CNT synthesis by fitting a quadratic surface, which is the common tool for process optimization. By solving the regression equation at desired values of the process responses as the optimization criteria, the optimum values of the chosen variables were obtained. Each parameter was coded at five levels: −α, −1, 0, +1, and +α (α = ±2). The range and the level of the variable in coded units from RSM investigations are presented in Table 1.

## 3. Results and Discussion

### 3.1. Suitable Dispersion Method

**Figure 3:**
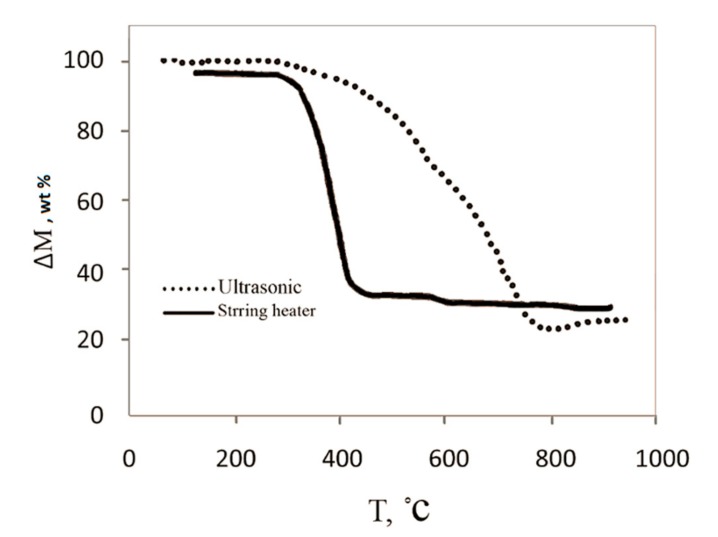
TGA data for pristine CNTs by using ultrasonic bath and stirring heater as two dispersion methods (catalyst concentration = 4 wt %, hydrogen flow = 200 cm^3^/min, and temperature = 1000 °C).

In order to select the effective dispersion method (stirring heater or ultrasonic bath) to obtain a homogeneous ferrocene-xylene solution during the synthesis process, the same operating conditions (catalyst concentration = 4 wt %, hydrogen flow = 200 cm^3^/min, and temperature = 1000 °C) were applied for each dispersion method. In another investigation, Bai et al. [4] reported that the inner diameter of CNTs is controlled by the size of the catalyst particle during the growth process. Similarly, in this study, controlling the size of catalyst particles and having a homogeneous solution had strong effect on the structure of synthesized CNTs. As displayed in Figure 3, the ultrasonic curve in contrast to stirring heater has a much sharper slope in the range of 400–700 °C, which corresponds to CNTs. 

Table 2 shows the percentage of each carbon structure existing in the sample using Figure 3 and indicates higher productivity yield of CNTs using ultrasonic bath in comparison with a stirring heater. When the ultrasonic bath is used, there would be less agglomeration of catalyst particles, smaller ferrocene particles size, and hence higher quality nanotubes and less amorphous carbon.

**Figure 4:**
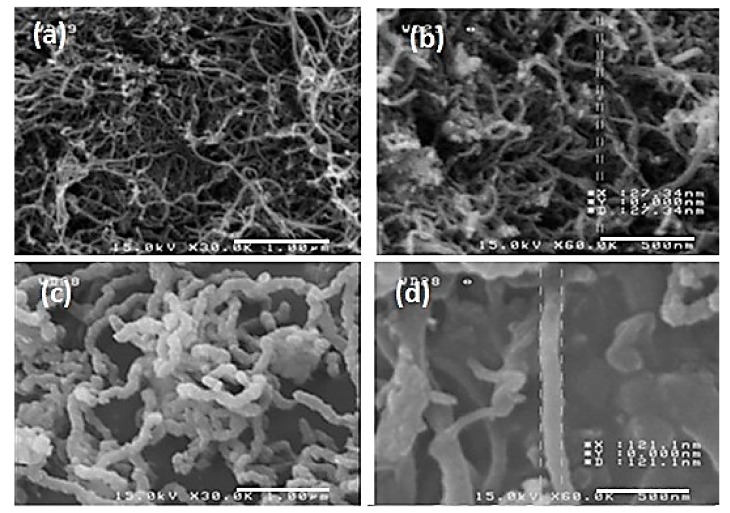
SEM image of pristine CNTs (**a**,**b**) using ultrasonic bath (the scale bars represent 1 µm and 500 nm, respectively) and (**c**,**d**) using stirring heater (the scale bars represent 1 µm and 500 nm, respectively) (catalyst concentration = 4 wt %, hydrogen flow = 200 cm^3^/min, and temperature = 1000 °C).

This is a clear indication of higher CNT production rate via ultrasonic bath application in the CNT synthesis. Moreover, the SEM results shown in Figure 4 reveal that using ultrasonic bath provides nanotubes with smaller diameters, which we consider beneficial results. As illustrated in Figure 4, the ultrasonic-assisted technique seems to produce CNTs with smaller diameters and less amorphous carbon in comparison with those produced using stirring heater (27 nm in comparison with 121 nm, respectively).

### 3.2. Central Composite Design and Fitted Regression Model

In order to study the relationship between the experimental parameters and Raman spectroscopy analysis of CNTs, 20 experiments designed by CCD were performed. Determination of the error value was the reason for repeating the experiments. Moreover, when the average of the data is used to estimate the effect of each factor, repetition makes the effect more accurate. The number of these repetitions is based on statistical rules and the method we chose for experimental design (central composite design). To put it more simply, the software determined the number of repetitions.

In this study, the diameter of the quartz tube and the reaction time were kept constant (2.5 cm and 80 min, respectively). The operating conditions of synthesis experiments and (*I*_G_*/I*_D_) ratio are given in Table 3. 

**Figure 5:**
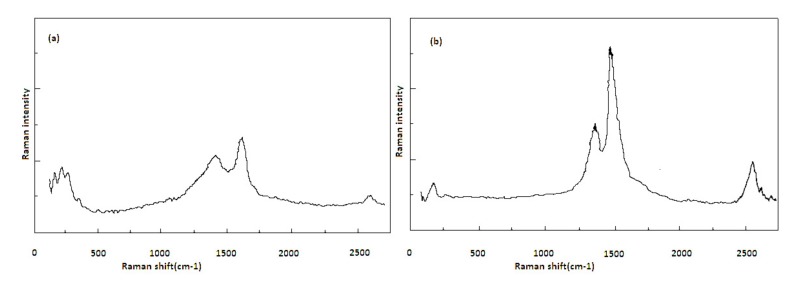
Raman spectra of the products using: (**a**) catalyst concentration = 5 wt %, hydrogen flow rate = 240 cm^3^/min, and temperature = 1100 °C, (*I*_G_*/I_D_*) = 1.87 and (**b**) catalyst concentration = 5 wt %, hydrogen flow rate = 240 cm^3^/min, and temperature = 900 °C, (*I*_G_*/I*_D_) = 2.5.

Moreover, in Figure 5 the Raman spectroscopy results of CNT samples with run number of (a) 4 and (b) 16 are shown.

Considering the operational conditions of these two samples, it is possible to find more insights on the temperature effect on the characteristics of synthesized nanotubes. Moreover, according to Figure 5, the value of (*I*_G_*/I*_D_) is higher at lower temperatures and decreases as the temperature increases. 

The empirical relationship between *I*_G_*/I*_D_ ratio (Y), where (*I*_G_*/I*_D_) is the ratio of symmetry-allowed G-band to disorder-induced D-band, and the synthesis parameters, in coded units and obtained by RSM, are shown in Equation (2). A group of peaks around 1340 cm^−1^, called the ‘‘D-band’’, is attributed to disorder in graphitic materials. Another significant mode is the G mode (G from graphite), which is in the range of 1550–1600 cm^−1^. G mode, which is present in most graphite-like materials, is associated with the planar vibrations of carbon atoms. The (*I*_G_*/I*_D_) ratio quantifies the structural quality of CNTs. Study of the D- and G-band modes by Raman spectroscopy gives information about the crystal structure of the product and about many of its interesting physical properties. A line is present around 2600 cm^−1^; it is an overtone or second-order harmonic of the D mode. It is called Gʹ or D* or 2D. The G’-band indicates long range order in a sample and arises from a two-phonon, second-order scattering process, which creates an inelastic phonon [33]. Longer 2D peaks show better crystallinity and graphitization, which means CNTs of better quality.

Raman spectroscopy gives information about purity, tube alignments, crystalline size, clustering of the sp^2^ phase within a given sample, the presence of sp^3^ hybridization and chemical impurities, its mass density, optical energy gap, elastic constants, and doping and defects of CNTs [34].

Moreover, A, B, and C are the coded values for catalyst concentration, hydrogen flow rate, and reaction temperature, respectively.

Y = (*I*_G_*/I*_D_) = +6.25 − 0.27A − 0.72B − 0.46C − 0.75A^2^ − 0.99B^2^ − 1.21C^2^ + 0.044AB + 0.13AC + 0.24BC (2)

F- and *p*-values, which are shown in Table 4, determine the significance of each coefficient. The model F-value of 60.71 shows that the model is significant. The *p*-values less than 0.05 indicate that the model terms are significant. Furthermore, *p*-value of hydrogen flow is <0.0001, which reveals that this parameter has a more significant influence on the quality of CNTs. The lack-of-fit value in this model (0.2532) shows a good compatibility between the experimental and the predicted data.

**Figure 6:**
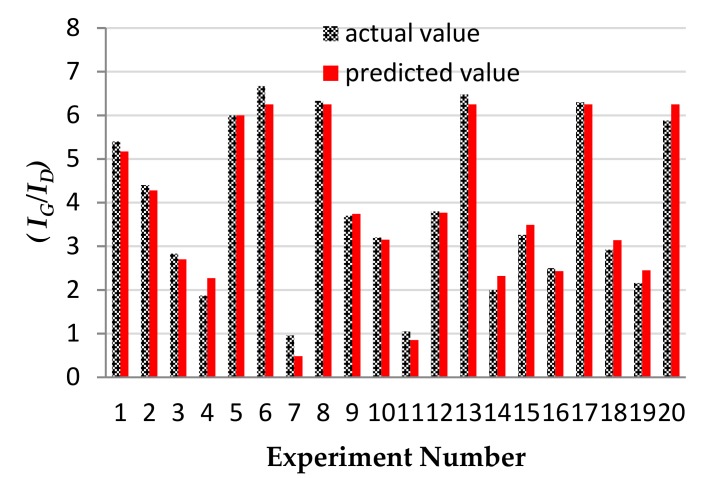
Experimental and theoretically predicted values for Raman spectroscopy analysis.

In this design, the value of the correlation coefficient, R^2^ = 0.982, and the value of the adjusted determination coefficient, R^2^ (Adj.) = 0.966, certify a good correlation between the predicted and the observed values. In order to obtain a better view of the model significance, the actual and the predicted values are compared in Figure 6.

### 3.3. Effect of Operating Conditions on CNTs 

**Figure 7:**
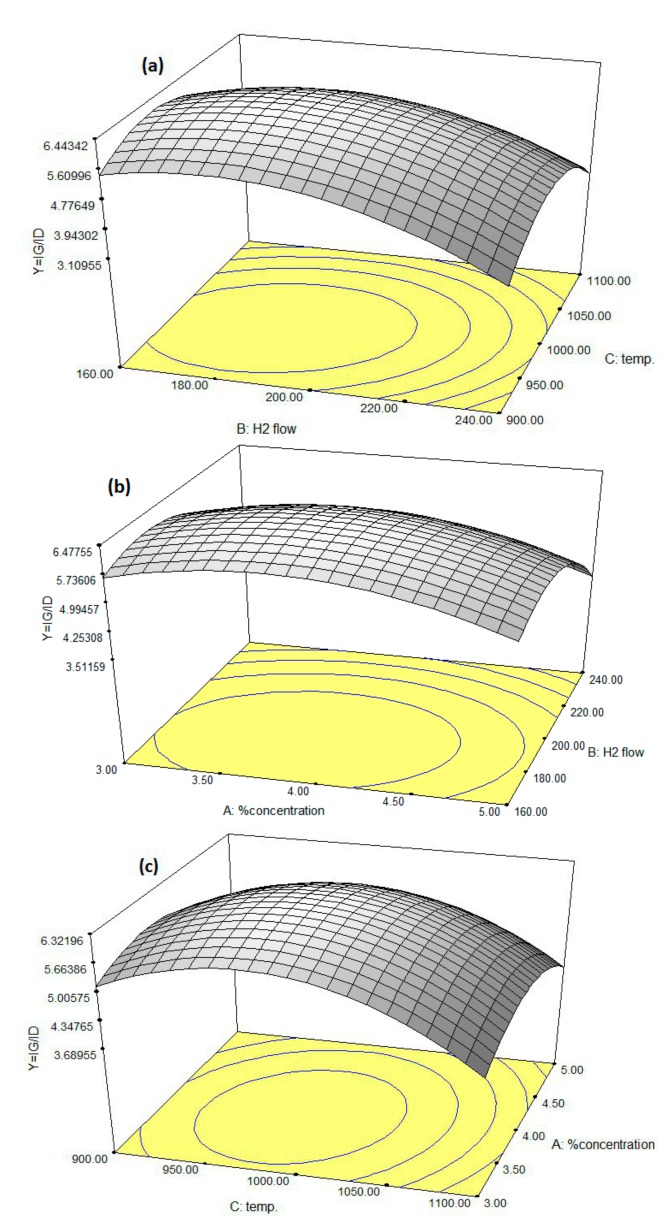
Three-dimensional surface graph to investigate the effect of (**a**) hydrogen flow rate (cm^3^/min) and reaction temperature (°C); (**b**) catalyst concentration (wt %) and hydrogen flow rate; (**c**) catalyst concentration (wt %) and reaction temperature (°C).

As shown in Figure 7, the effects of operating conditions (reaction temperature, hydrogen flow rate, and catalyst concentration) on CNT synthesis were investigated. The trend of hydrogen in the diagrams is very complicated. First, it acts as the carrier gas. The hydrogen flow carried xylene and catalyst precursors (ferrocene) into the reactor, and the hydrogen flow rate affects the partial pressure of xylene and ferrocene. Second, ferrocene is reduced and decomposed into iron atoms in the hydrogen, which further agglomerate into iron clusters or iron particles [18]. The results of Figure 7a show that by increasing the hydrogen flow rate, (*I*_G_*/I*_D_) value increases with a low slope up to approximately 180 cm^3^/min of hydrogen flow in which the maximum quality of CNTs is produced. Subsequently, further increase in hydrogen flow contributes to a decrease in CNT quality. As illustrated in Figure 7b, the (*I*_G_*/I*_D_) ratio improves slightly by increasing the catalyst concentration. However, this ratio decreases with a low slope after the optimum point (the maximum value of (*I*_G_*/I*_D_)). As displayed in Figure 7c, the higher reaction temperature improves the quality of CNTs up to approximately 970 °C at which the highest quality of CNTs is observed, and increasing the temperature beyond that point decreases CNT quality. This phenomenon may be explained by considering that increasing the temperature with a constant catalyst concentration first enhances the solubility of carbon atoms into iron particles up to the optimum point (the maximum value of (*I*_G_*/I*_D_)) [8]. Beyond this point, a decrease in CNT quality occurs, which is due to the increase in agglomeration of iron nanoparticles after melting. Decreasing the concentration of iron particles in the reaction volume decreases the number of collisions among these species, hence, the resulting nuclei are relatively small and uniform. This phenomenon results in preferential formation of SWNTs. Increasing the concentration of Fe particle contributes to the formation of larger catalytic sites and products consisting primarily of MWNTs [9]. The effect of catalyst concentration on CNT synthesis was studied in the operating range of 3–5 wt %. The higher ferrocene concentrations enhance CNT quality up to approximately 4 wt % at which the best quality of CNTs is formed but, beyond that, a decrease in CNT quality (*I*_G_*/I*_D_) is observed. The observed trend may be explained by considering that a higher agglomeration tendency is expected at higher ferrocene concentrations. The explained trends (relative symmetry) are also noticeable via the circular contours in Figure 7. The higher observed symmetry in Figure 7c displays a more uniform behavior of catalyst concentration during the CNT synthesis compared with that of hydrogen flow rate and reaction temperature.

**Figure 8:**
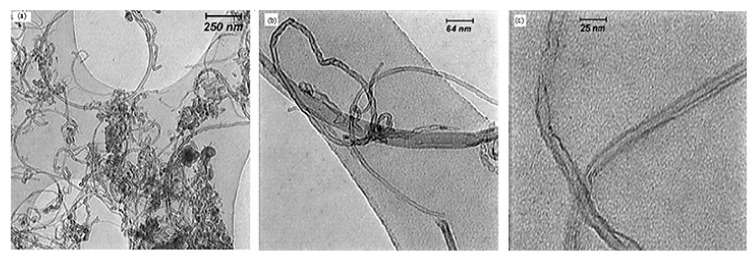
TEM images of as-prepared CNTs (**a**) catalyst concentration = 3 wt %, hydrogen flow = 240 cm^3^/min, and temperature = 900 °C; (**b**) catalyst concentration = 4 wt %, hydrogen flow = 120 cm^3^/min, and temperature = 1000 °C; (**c**) catalyst concentration = 5 wt %, hydrogen flow = 160 cm^3^/min, and temperature = 900 °C, by using ultrasonic bath.

TEM images from a number of as-prepared CNTs are shown in Figure 8. The results presented in Figure 8a–c are associated with the tests 10, 9, and 2, respectively. As it is observed in Figure 8, the diameter of nanotubes ranges between 10 and 25 nm and CNTs are uniform. 

### 3.4. Optimization Using the Desirability Functions

**Figure 9:**
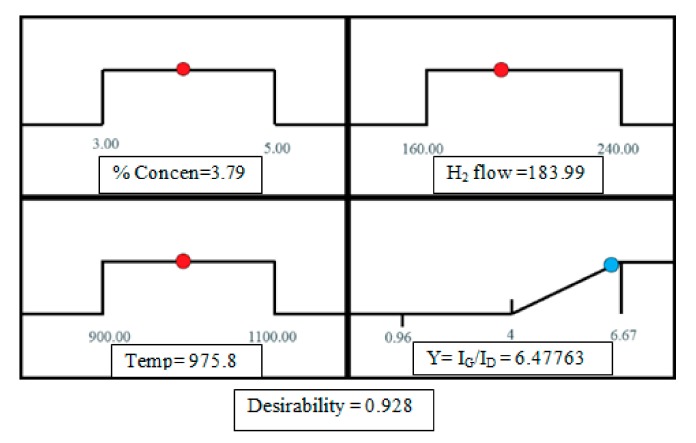
Desirability ramp for numerical optimization of three independent variables (catalyst concentration ratio, hydrogen flow rate, reaction temperature) and response surface (CNT production).

The optimization was carried out by Design Expert aiming to find the best operating conditions that will lead to the best quality CNTs. We specified a limit for each factor. Desirability in the program is defined to be an objective function ranging from zero (outside the limits) to one (at the goal). The maximization of this function is the ultimate aim of this program. Since the response surfaces have a curvature format, more than one maximum point and their combination into the desirability function is expected. The standard routine of this computer software is to start from many points in the design space until the search ends up by finding the “best” local maximum. A multiple response method was used for optimizing any combination of four goals, namely, the catalyst concentration ratio, reaction temperature, hydrogen flow rate, and CNT quality (*I*_G_*/I*_D_). An optimum point which maximizes the desirability function was found by the numerical optimization. Figure 9 shows a ramp desirability generated from 10 optimum points via numerical optimization. 

**Figure 10:**
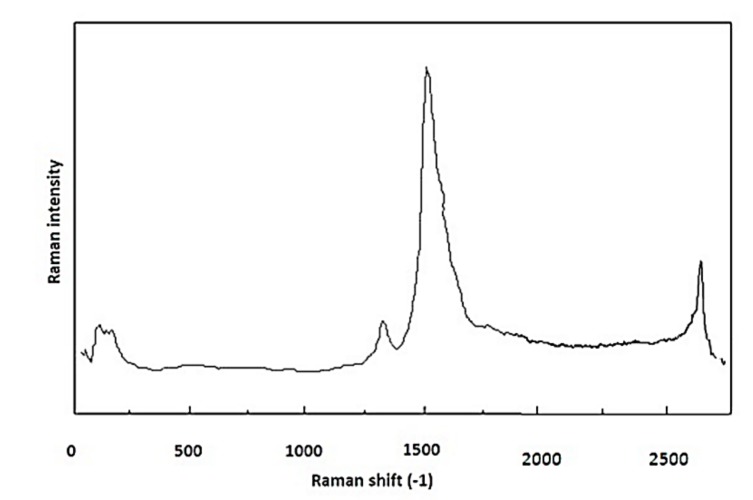
Raman spectra of the CNTs synthesizing in optimum conditions (catalyst concentration = 3.79 wt %, hydrogen flow = 185 cm^3^/min, and temperature = 975 °C, (*I*_G_*/I*_D_) = 6.4)).

Based on the results from Figure 10, the best local maximum response surface ((*I*_G_*/I*_D_) = 6.47) was predicted via RSM (Equation (2)) to be at the optimal operating conditions of catalyst concentration ratio of 3.8 wt %, hydrogen flow rate of 184 mL/min, reaction temperature of 976 °C, and desirability of 0.93. The triplicate experimental syntheses were carried out at the aforementioned optimum operating conditions in which a very close compatibility (CNTs production (*I*_G_/*I*_D_) = 6.4) was observed. As shown in Figure 10, (*I*_G_*/I*_D_) is close to the predicted value.

According to Figure 10, higher quality CNTs are produced at optimal operating conditions. The radial breathing mode (RBM) Raman features are unique to CNTs and happen with frequencies between 120 cm^−1^ and 350 cm^−1^ for SWNTs [35]. The presence of this peak in most of the samples is due to the impact of ultrasonic bath and its prevention of the agglomeration of iron nanoparticles, which has increased the probability of producing CNTs with smaller diameters. Considering that in the sample produced under the optimum conditions we have the lowest D-band value in comparison with G-band value, it can be concluded that the highest number of few-walls were produced in the sample. 

## 4. Conclusions

In the present study, in order to control the size of CNT clusters and distribute them uniformly, ultrasonic bath was used in the floating catalyst technique. The obtained results show the effectiveness of this method in controlling catalyst nanoparticle size, decreasing the produced amorphous carbon, and improving the quality of produced CNTs in comparison with the previous methods.

To this end, RSM was applied in characterizing the effects of this parameter and the three process variables in order to consider the interaction effects, too. Catalyst concentration, hydrogen flow rate, and synthesis temperature, which are the three effective parameters in CCD, were optimized in the present work. Analysis results showed that hydrogen flow rate and temperature are more effective than catalyst concentration. This might have been induced by the fact that the experiments were carried out at low catalyst concentration.

The quadratic model obtained by CCD and the lack-of-fit value in this model (0.2532) show a good compatibility between the experimental and the predicted data. In this design, analysis of variance displayed a coefficient of determination value (0.966–0.982) which ensures a good correlation between the predicted and the observed values. Furthermore, triplicate experimental synthesis at the optimum operating conditions resulted in a CNT production of 6.4, which confirms the modeling prediction.

## Figures and Tables

**Table 1:** Experimental ranges and levels of the independent variables.

Variables	Range and Level				
	−α	−1	0	1	+α
Catalyst concentration (wt %) (*x*_1_)	2	3	4	5	6
Reaction temperature (°C) (*x*_2_)	800	900	1000	1100	1200
Hydrogen flow (cm^3^/min) (*x*_3_)	120	100	200	240	280

**Table 2:** Mass percentage of carbon structures in pristine CNTs using ultrasonic bath and stirring heater.

Methods	Carbon Structure (wt %)		
	Amorphous Carbon	SWNT	MWNT
Ultrasonic bath	7	30	45
Stirring heater	65	7	8

**Table 3:** Experimental runs based on central composite design (CCD) and Raman spectroscopy data at constant reaction time (80 min) and internal diameter (2.5 cm).

Run No.	Catalyst Concentration (wt %)	Hydrogen Flow Rate (cm^3^/min)	Temperature (°C)	Raman (*I*_G_*/I*_D_**)
1	3	160	900	5.4
2	5	160	900	4.4
3	6	200	1000	2.83
4	5	240	1100	1.87
5	4	200	1000	6
6	4	200	1000	6.67
7	4	200	1200	0.96
8	4	200	1000	6.33
9	4	120	1000	3.7
10	3	240	900	3.2
11	4	280	1000	1.05
12	2	200	1000	3.8
13	4	200	1000	6.48
14	4	200	800	2.00
15	3	160	1100	3.26
16	5	240	900	2.5
17	4	200	1000	6.3
18	5	160	1100	2.92
19	3	240	1100	2.16
20	4	200	1000	5.88

**Table 4:** Analysis of variance (ANOVA) for the response surface quadratic model.

Source	Sum of Square	DF	Mean Square	F-Value	*p*-Value	Remarks
Model	68.11	9	7.57	60.71	<0.0001	significant
A	1.14	1	1.14	9.14	0.0128	
B	8.34	1	8.34	66.89	<0.0001	
C	3.39	1	3.39	27.23	0.0004	
A2	14.29	1	14.29	114.63	<0.0001	
B2	24.59	1	24.59	197.23	<0.0001	
C2	36.97	1	36.97	296.59	<0.0001	
AB	0.015	1	0.015	0.12	0.7332	
BC	0.48	1	0.48	3.81	0.0794	
AC	0.14	1	0.14	1.15	0.3091	
Residual	1.25	10	0.12			
Lack of fit	0.81	5	0.16	1.88	0.2532	not significant
Pure error	0.43	5	0.087			
Cor total	69.36	19				

CV: 9.09; SD: 0.35; R^2^: 0.982; R^2^ (Adj.): 0.966.

## References

[1] Gibson C.T., Carnally S., Roberts C.J. (2007). Attachment of carbon nanotubes to atomic force microscope probes. Ultramicroscopy.

[2] Kumar S., Rani R., Dilbaghi N., Tankeshwar K., Kim K.-H. (2017). Carbon nanotubes: A novel material for multifaceted applications in human healthcare. Chem. Soc. Rev..

[3] Grace T., Yu L., Gibson C., Tune D., Alturaif H., Al Othman Z., Shapter J. (2016). Investigating the effect of carbon nanotube diameter and wall number in carbon nanotube/silicon heterojunction solar cells. Nanomaterials.

[4] Bai S., Li F., Yang Q., Cheng H.-M., Bai J. (2003). Influence of ferrocene/benzene mole ratio on the synthesis of carbon nanostructures. Chem. Phys. Lett..

[5] Liu Z., Robinson J.T., Tabakman S.M., Yang K., Dai H. (2011). Carbon materials for drug delivery & cancer therapy. Mater. Today.

[6] Díaz-Flores P., Arcibar-Orozco J., Perez-Aguilar N., Rangel-Mendez J., Medina V.O., Alcalá-Jáuegui J. (2017). Adsorption of organic compounds onto multiwall and nitrogen-doped carbon nanotubes: Insights into the adsorption mechanisms. Water Air Soil Pollut..

[7] Ahn C.H., Baek Y., Lee C., Kim S.O., Kim S., Lee S., Kim S.-H., Bae S.S., Park J., Yoon J. (2012). Carbon nanotube-based membranes: Fabrication and application to desalination. J. Ind. Eng. Chem..

[8] Moisala A., Nasibulin A.G., Kauppinen E.I. (2003). The role of metal nanoparticles in the catalytic production of single-walled carbon nanotubes—A review. J. Phys. Condens. Matter.

[9] Fan Y.-Y., Kaufmann A., Mukasyan A., Varma A. (2006). Single-and multi-wall carbon nanotubes produced using the floating catalyst method: Synthesis, purification and hydrogen up-take. Carbon.

[10] José-Yacamán M., Miki-Yoshida M., Rendon L., Santiesteban J. (1993). Catalytic growth of carbon microtubules with fullerene structure. Appl. Phys. Lett..

[11] Nikoo M.K., Saeidi S., Lohi A. (2015). A comparative thermodynamic analysis and experimental studies on hydrogen synthesis by supercritical water gasification of glucose. Clean Technol. Environ. Policy.

[12] Saeidi S., Fazlollahi F., Najari S., Iranshahi D., Klemeš J.J., Baxter L.L. (2017). Hydrogen production: Perspectives, separation with special emphasis on kinetics of WGS reaction: A state-of-the-art review. J. Ind. Eng. Chem..

[13] Saeidi S., Amin N.A.S., Rahimpour M.R. (2014). Hydrogenation of CO_2_ to value-added products—A review and potential future developments. J. CO_2_ Util..

[14] Saeidi S., Najari S., Fazlollahi F., Nikoo M.K., Sefidkon F., Klemeš J.J., Baxter L.L. (2017). Mechanisms and kinetics of CO_2_ hydrogenation to value-added products: A detailed review on current status and future trends. Renew. Sustain. Energy Rev..

[15] McKee G.S., Deck C.P., Vecchio K.S. (2009). Dimensional control of multi-walled carbon nanotubes in floating-catalyst cvd synthesis. Carbon.

[16] Othman R.N., Kinloch I.A., Wilkinson A.N. (2013). Synthesis and characterisation of silica-carbon nanotube hybrid microparticles and their effect on the electrical properties of poly (vinyl alcohol) composites. Carbon.

[17] Li Q., Yan H., Zhang J., Liu Z. (2004). Effect of hydrocarbons precursors on the formation of carbon nanotubes in chemical vapor deposition. Carbon.

[18] Yamada T., Namai T., Hata K., Futaba D.N., Mizuno K., Fan J., Yudasaka M., Yumura M., Iijima S. (2006). Size-selective growth of double-walled carbon nanotube forests from engineered iron catalysts. Nat. Nanotechnol..

[19] Dang F., Enomoto N., Hojo J., Enpuku K. (2010). Sonochemical coating of magnetite nanoparticles with silica. Ultrason. Sonochem..

[20] Yan J., Xu Z., Shi L., Ma X., Yang S. (2011). Ultrasonic assisted fabrication of particle reinforced bonds joining aluminum metal matrix composites. Mater. Des..

[21] Beltowska-Lehman E., Indyka P., Bigos A., Szczerba M.J., Kot M. (2015). Ni-W/ZrO_2_ nanocomposites obtained by ultrasonic dc electrodeposition. Mater. Des..

[22] Versteeg V.A., Avedisian C.T., Raj R. (1995). Method and Apparatus for CVD Using Liquid Delivery System with an Ultrasonic Nozzle. U.S. Patent,.

[23] Saeidi S., Jouybanpour P., Mirvakilli A., Iranshahi D., Klemeš J.J. (2016). A comparative study between modified data envelopment analysis and response surface methodology for optimisation of heterogeneous biodiesel production from waste cooking palm oil. J. Clean. Prod..

[24] Chen C.-M., Dai Y.-M., Huang J.G., Jehng J.-M. (2006). Intermetallic catalyst for carbon nanotubes (CNTs) growth by thermal chemical vapor deposition method. Carbon.

[25] Nariyan E., Sillanpää M., Wolkersdorfer C. (2018). Uranium removal from pyhäsalmi/finland mine water by batch electrocoagulation and optimization with the response surface methodology. Sep. Purif. Technol..

[26] Jiang W., Joens J.A., Dionysiou D.D., O’Shea K.E. (2013). Optimization of photocatalytic performance of TiO_2_ coated glass microspheres using response surface methodology and the application for degradation of dimethyl phthalate. J. Photochem. Photobiol. A Chem..

[27] Bai Y., Saren G., Huo W. (2015). Response surface methodology (RSM) in evaluation of the vitamin C concentrations in microwave treated milk. J. Food Sci. Technol..

[28] Dhawane S.H., Kumar T., Halder G. (2015). Central composite design approach towards optimization of flamboyant pods derived steam activated carbon for its use as heterogeneous catalyst in transesterification of hevea brasiliensis oil. Energy Convers. Manag..

[29] Habibi R., Rashidi A.M., Daryan J.T. (2010). Study of the rod-like and spherical nano-ZnO morphology on H_2_S removal from natural gas. Appl. Surf. Sci..

[30] Živković J., Šavikin K., Janković T., Ćujić N., Menković N. (2018). Optimization of ultrasound-assisted extraction of polyphenolic compounds from pomegranate peel using response surface methodology. Sep. Purif. Technol..

[31] Li J., Liu Q., qing Ji Q., Lai B. (2017). Degradation of *p*-nitrophenol (PNP) in aqueous solution by Fe^0^-PM-PS system through response surface methodology (RSM). Appl. Catal. B Environ..

[32] Ghorbani F., Younesi H., Ghasempouri S.M., Zinatizadeh A.A., Amini M., Daneshi A. (2008). Application of response surface methodology for optimization of cadmium biosorption in an aqueous solution by saccharomyces cerevisiae. Chem. Eng. J..

[33] Lehman J.H., Terrones M., Mansfield E., Hurst K.E., Meunier V. (2011). Evaluating the characteristics of multiwall carbon nanotubes. Carbon.

[34] Saito R., Hofmann M., Dresselhaus G., Jorio A., Dresselhaus M. (2011). Raman spectroscopy of graphene and carbon nanotubes. Adv. Phys..

[35] Dresselhaus M.S., Dresselhaus G., Saito R., Jorio A. (2005). Raman spectroscopy of carbon nanotubes. Phys. Rep..

